# *Bombyx mori* Pupae as a Novel Ingredient from an Underutilized Sericulture Product: A Dual Approach Based on Sustainable Extractions and Sample Pretreatment Strategies

**DOI:** 10.1021/acssuschemeng.5c10626

**Published:** 2026-01-09

**Authors:** Guilherme Dallarmi Sorita, Luca Tassoni, Alessio Saviane, Alejandro Cifuentes, Elena Ibáñez, Luana Cristina dos Santos

**Affiliations:** † Foodomics Laboratory, Institute of Food Science Research (CIAL) (CSIC-UAM), Nicolás Cabrera 9, Madrid 28049, Spain; ‡ Department of Chemical and Food Engineering, Federal University of Santa Catarina, Florianópolis 88040-900, Brazil; § Council for Agricultural Research and Economics—Research Centre for Agriculture and Environment, Via eulero, 6A, Padova 35143, Italy

**Keywords:** insect oil, green extraction, natural solvents, waste valorization, circular economy

## Abstract

*Bombyx mori* (*B. mori*) pupae, a major byproduct of the silk industry, can be upcycled as a source of bioactive lipids for food, nutraceutical, and cosmetic applications. This study aimed to sustainably recover the lipophilic fraction from*B. mori* using supercritical fluid extraction (SFE) assisted by natural hydrophobic solvents (SFE-NHSolv) as an alternative to Soxhlet (SOX) extraction with hexane while also evaluating the impact of two different pretreatment methods, freeze-drying (FD) and blanching (B), followed by FD (B+FD). The process yield, lipid and carotenoid profiles, bioactivities (antioxidant and antibacterial), and environmental performance of SOX and SFE-NHSolv were assessed. Among the processes, SFE-NHSolv with B+FD achieved the highest yield (55.98%) compared with Soxhlet (38.3%). The fatty acid profile revealed that the lipid fraction is mainly composed of linolenic, oleic, and palmitic acids. Five carotenoids were tentatively identified in SOX extracts including lutein, zeaxanthin, and β-carotene. Extracts exhibited higher antioxidant capacity by ABTS (3.25–15.44 μmol TE g^–1^) than by DPPH (2.59–7.23 μmol TE g^–1^), with SFE-NHSolv B+FD showing the highest value. No antibacterial activity was observed against *Staphylococcus aureus* and*Escherichia coli*. The sustainability assessment confirmed SFE-NHSolv as a cleaner and more eco-efficient process than SOX, capable of producing ready-to-use extracts. In summary, this study contributes to the advancement of sustainable extraction technologies. It promotes the sustainable use of silk industry byproducts, offering eco-friendly solutions for developing high-value ingredients for the food, nutraceutical, and cosmetic sectors.

## Graphical abstract

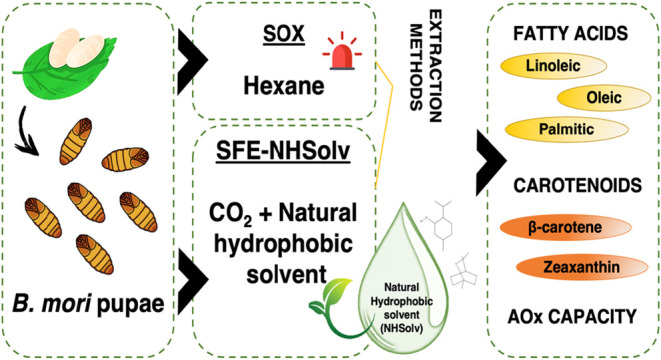

## 1 Introduction

The growing number of people seeking alternative and sustainable sources of nutrition has driven increased interest in functional edible oils, especially in the wake of recent global disruptions, such as the COVID-19 pandemic and the Ukraine war, which have underscored the need to identify alternative food sources.^1^ Traditionally, animal lipids are obtained from dairy, meat, fish, and seafood, while plant-based lipids are sourced from seeds, nuts, avocados, and cocoa. More recently, insects have emerged as a promising sustainable alternative due to their energy-rich and nutritionally valuable lipid profiles.^2,3^ Their short lifecycles, rapid growth rates, and nutritional composition make edible insects viable alternatives to conventional agricultural and animal husbandry products.^2^

Silkworm (*Bombyx mori*) pupae are a promising insect resource and represent a major byproduct of the silk industry, accounting for approximately 60% of the cocoon’s total weight.^2–4^ Although often discarded or used as fertilizer and animal feed, silkworm pupae are rich in proteins, amino acids, lipids, vitamins, and minerals, positioning them as a valuable resource for human nutrition.^2–4^ Notably, *B. mori* pupae are primarily composed of fatty acids and proteins, with their fatty acid profile mainly composed of unsaturated fatty acids, particularly polyunsaturated fatty acids, such as linoleic acid and linolenic acid, which are recognized for their high nutritional value.^2^

Before being processed for the recovery of valuable components, proper pretreatment of insects is essential to ensure microbiological safety and improve the efficiency of subsequent steps. Among the available methods, blanching (using hot water or steam) and freeze-drying are the most applied, either individually or in combination.^5,6^ Blanching, typically performed with hot water or steam, inactivates enzymes and microorganisms, reduces spoilage, and facilitates drying. Despite potentially affecting some physical and chemical properties, this step enhances the material stability and processability.^6^ Freeze-drying, on the other hand, removes water through sublimation, preserving nutrients, bioactive compounds, and sensory qualities. Freeze-drying yields a lightweight, porous, and stable product but requires careful packaging due to its hygroscopic nature and involves higher operational costs.^6^ Evaluating these pretreatment impacts on the raw material is crucial for optimizing extraction efficiency and maintaining the integrity of target compounds.

Following pretreatment, the choice of an appropriate extraction method is crucial for maximizing yield and preserving compound quality. As an efficient and sustainable alternative to conventional oil extraction methods, supercritical fluid extraction (SFE) has gained increasing attention. Among supercritical solvents, carbon dioxide (CO_2_) is particularly attractive due to its low toxicity, mild critical conditions, and ease of removal from the final product.^7^ The incorporation of food-grade cosolvents is an important strategy to enhance the solvation power toward more polar compounds, impacting the overall process efficiency and enabling the recovery of high-value compounds as clean, and ready-to-use extracts.^8^ This technology aligns with the principles of green chemistry. It supports the United Nations Sustainable Development Goal 12 (Responsible Consumption and Production), providing safer, more sustainable, and environmentally friendly solutions for the food, nutraceutical, and cosmetic industries.^9^

To the best of our knowledge, a few studies have systematically evaluated the use of supercritical fluid extraction assisted by natural-based food-grade hydrophobic cosolvents (SFE-NHSolv) for the recovery of the lipid fraction from *B. mori* pupae and compared its performance with conventional Soxhlet extraction. Yet, to the best of our knowledge, the combined effects of different pretreatment strategies (freeze-drying vs blanching followed by freeze-drying) on extraction efficiency, lipid quality, carotenoid composition, and biological activities have not yet been comprehensively investigated. In addition, environmental assessments of SFE applied to insect-derived matrices are scarce in the literature. The purpose of this study is to establish a clean, efficient, and environmentally responsible pathway for the valorization of *B. mori* pupae into high-value functional lipid ingredients for industrial applications.

In this context, this study aimed to recover the lipid fraction from *B. mori*, an agro-industrial byproduct, through supercritical fluid extraction (SFE) assisted by food-grade natural-based hydrophobic solvents (SFE-NHSolv) as a sustainable alternative to conventional Soxhlet extraction (SOX). The influence of two different pretreatment methods, freeze-drying and blanching followed by freeze-drying, was also considered. Process yield, fatty acid profile, carotenoid composition, and antioxidant and antibacterial activities were evaluated along with the environmental performance of SOX and SFE-NHSolv processes. This comprehensive evaluation offers scientific advances in sustainable extraction and byproduct valorization while contributing to the development of safer, cleaner, and ready-to-use functional ingredients for food, nutraceutical, and cosmetic applications, promoting both environmental benefits and societal well-being.

## 2 Materials and Methods

### 2.1 Materials

For the natural-based hydrophobic solvent (NHSolv) preparation, eucalyptol (1,8-cineole, CAS: 470-82-6, >98% purity) and menthol (CAS: 89-78-1, >98% purity) were purchased from TCI Chemicals (Tokyo). For ABTS (2,2′-azino-bis-3-ethylbenzthiazoline-6-sulfonic acid) assay, potassium persulfate (K_2_S_2_O_8_, 99% purity) was acquired from Montplet & Estaban SA (Madrid, Spain), ABTS (98% purity) and potassium dihydrogen phosphate (K_2_HPO_4_, >98% purity) from Sigma-Aldrich (Steinheim, Germany), and sodium hydrogen phosphate anhydrous (Na_2_HPO_4_, >99% purity) from Merck (Darmstadt, Germany). DPPH (1,1-diphenyl-2-picrylhydrazyl, >97% purity) was purchased from TCI Chemicals (Tokyo, Japan). Trolox (6-hydroxy-2,5,7,8-tetramethylchroman-2-carboxylic acid, >98% purity), used as the standard reference for ABTS and DPPH methods, was purchased from Sigma-Aldrich (Steinheim, Germany). β-Carotene standard (>98% purity) for HPLC analysis was obtained from Sigma-Aldrich (Steinheim, Germany).

### 2.2 Methodology

Figure 1 illustrates the methodological procedure adopted in this study, and the experimental assays are detailed in the following sections.

**Figure 1:**
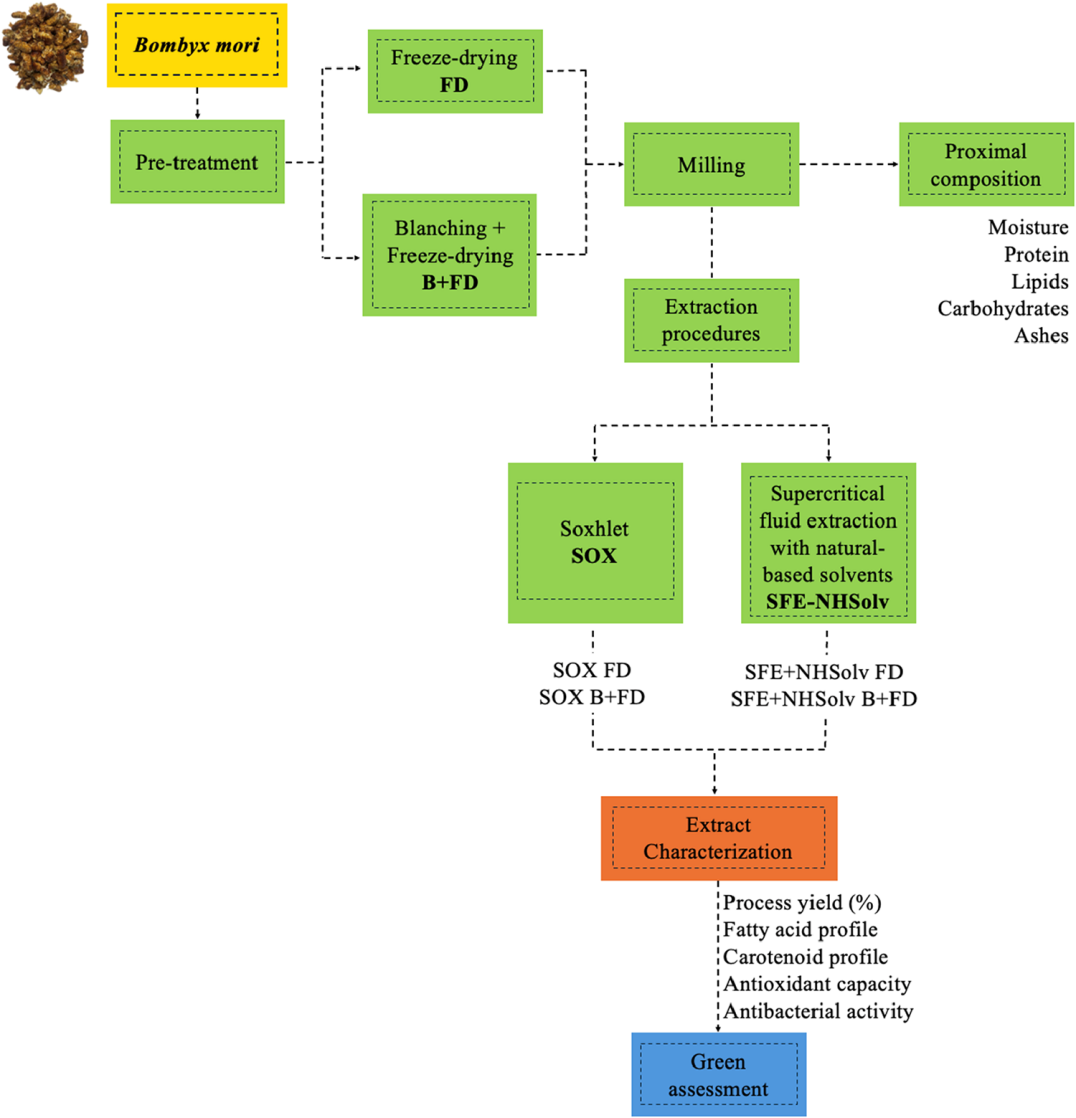
Schematic workflow illustrating the methodological procedures employed in this study.

#### 2.2.1 Natural-Based Solvents Synthesis and Characterization

The solvents used in this study were selected based on previous reports highlighting their effectiveness in extracting hydrophobic compounds.^10^ The mixtures were prepared by combining menthol (Me) and eucalyptol (Eu) in a 1:1 molar ratio (w/w). The natural-based solvent system was heated to 70 °C and continuously stirred until a homogeneous liquid was obtained. After 24 h, the mixture reached stability, with no visible solid precipitation. Additionally, the solvent’s density and pH were experimentally determined at 25 °C, yielding values of 0.90159 g cm^–3^ and 5.2, respectively.

#### 2.2.2 *B. mori* Pupae: Preliminary Selection, Pretreatment, and Proximate Composition

*B. mori* larvae were reared in the Veneto region (North-East Italy) by a local farmer. All of the silkworms used for this study came from a single farm to minimize the effect that different rearing conditions might have had on pupal composition. Larvae were fed fresh mulberry leaves three times per day during the feeding periods, while the meals were suspended during the molting phase. Mulberry leaves were administered fresh and dry in the morning, at noon, and in the afternoon. The cocoons that did not meet sericulture standards were manually discarded. Subsequently, the pupae were removed from the selected cocoons and divided into two groups: one underwent blast chilling at −40 °C followed by freeze-drying (FD) using a freeze-dryer (Gelert CryoDryer5, Langweid a. Lech, Germany), while the other was subjected to blanching at 100 °C for 4 min, followed by blast chilling at −40 °C and subsequent freeze-drying (B+FD). The two sample groups (FD and B+FD) of *B. mori* pupae were milled to reduce particle size and stored at −20 °C prior to analysis. Chemical proximate composition was estimated according to their moisture at 105 °C in a DAB moisture analyzer (Kern), while ashes and lipids were obtained according to official methods 972.15 and 963.15 from AOAC (1997). The protein content was determined using the Kjeldahl method, with a conversion factor specifically corrected for insects of 4.76, as recommended by Janssen et al.,^11^ to avoid overestimation caused by nitrogen contributions from chitin.

#### 2.2.3 Lipid Extraction of *B. mori* Pupae

##### 2.2.3.1 Conventional Extraction: Soxhlet

Soxhlet extraction was performed as described in Sorita et al.^12^ Briefly, 150 mL of hexane was recirculated over 3 g of raw material in a Soxhlet apparatus for 8 h at the solvent’s boiling temperature. After extraction, residual hexane was removed by evaporation under a nitrogen stream using a TurboVap LV instrument (Caliper, Biotage AB, Uppsala, Sweden). The recovered extracts were stored at −20 °C in the absence of light until analysis. Extraction yield (wt %) was calculated as the ratio between the mass of the recovered extract and the mass of the raw material used in the extraction. Results are expressed as the mean ± standard deviation from the duplicate experiments.

##### 2.2.3.2 Supercritical CO_2_+Me:Eu Liquid Mixture as Cosolvent

A custom-built, lab-scale supercritical fluid extraction (SFE) system was employed to recover lipophilic extracts from*B. mori*, following the methodology previously established by our research group.^13–15^ To ensure stable and reproducible operating conditions, the extraction apparatus was assembled using a liquid pump (PU-2080, Jasco, Tokyo, Japan), a high-pressure CO_2_ pump (PU-2080 Plus CO_2_; Jasco, Hachioji, Japan), a homemade oven, and a manual back-pressure regulator (Vici-Valco Instruments Co., Inc., Houston). Briefly, approximately 1 g of the sample and 20 g of glass beads (used as a dispersing agent) were loaded into a 25 mL stainless steel extraction cell. Glass wool was inserted at both ends of the extraction cell. A layer of glass beads was placed at the bottom near the solvent inlet to assist in solvent dispersion. The extraction was conducted at a constant flow rate of 4 mL min^–1^ (measured at the head pump), using supercritical CO_2_ with 15% (v/v) cosolvent (Me:Eu 1:1 w/w). The operating temperature and pressure were maintained at 60 °C and 200 bar, respectively. An extraction time of 90 min was selected. The SFE conditions applied in this study were selected based on previously reported protocols that demonstrated high extraction efficiency for lipid-rich insect biomasses.^16,17^ Recent results from our research group,^17^ obtained under the same operating conditions, showed excellent lipid recovery from *Galleria mellonella* larvae. Although species-specific differences may occur, *G. mellonella* and *B. mori* pupae share comparable matrix characteristics, particularly with respect to the lipid content, moisture, and cuticular structure, supporting the suitability of these parameters for *B. mori*. Following each extraction, the system was flushed under the same conditions to recover any residual extract from the tubing. Extract yield was determined by drying an aliquot of the collected extract at 90 °C under a constant nitrogen flow. Aliquots were repeatedly weighed and subjected to evaporation until a constant mass was achieved, ensuring the removal of volatile compounds. The yield was calculated as the ratio between the mass of the dried extract and the mass of the raw material used for extraction. Additionally, the characteristic aroma of menthol and other volatile cosolvents was no longer perceptible in the final extract. After extraction, all extracts were immediately wrapped in aluminum foil to prevent light exposure and stored at −20 °C to minimize degradation prior to analytical characterization. Results are expressed as the mean ± standard deviation from duplicate experiments.

#### 2.2.4 Chemical Profiling of Extracts

##### 2.2.4.1 Lipid Profile by GG-MS

The fatty acid methyl esters (FAMEs) profile of *B. mori* lipid extracts was analyzed using a GC-MS system Shimadzu GC 2010 equipped with a Shimadzu AOC-20i autosampler and a split/splitless injector coupled to a QP-2010 Plus single quadrupole mass spectrometer (Shimadzu Corporation, Kyoto, Japan). First, the derivatization method followed the proposed method described by Golmakani et al.^18^ In summary, 3 mL of methanolic acetyl chloride solution (95:5, v/v) was added to approximately 30 mg of lipid extracts obtained from each extraction method, which also contained 2 mg of internal standard (heptadecanoic acid). The acid-catalyzed reaction took place at 85 °C for 1h. After that, the solution was cooled down to room temperature, 1 mL of ultrapure water was added, the mixture was vigorously shaken for 1 min, and 3 mL of hexane (containing 0.01% BHT) was then added to separate FAMEs from aqueous fraction. The hexane layer was diluted (1:1, v/v) and transferred to a 2 mL vial and injected into GC-MS. The GC-MS conditions were as follows: a ZB-WAX fused silica capillary column (30 m × 0.25 mm i.d., 0.25 μm film thickness; Phenomenex, Torrance) was used for the separation of FAMEs. Injector, interface, and ionization chamber temperatures were set at 260, 245, and 250 °C, respectively. The oven temperature was started at 80 °C (2 min hold), followed by consecutive ramps of 20 °C min^–1^ until 180 °C (2 min hold), 4 °C min^–1^ until 207 °C (3 min hold), and finally 4 °C min^–1^ to 220 °C. A volume of 0.5 μL of sample was injected in split mode (1:50). Helium served as the carrier gas at a column flow of 1.44 mL min^–1^. A 3.7 min solvent delay was applied to the mass spectrometer. Compounds were detected in scan mode (40–400 *m*/*z*) and identified using updated libraries from Wiley and NIST, confirming the fragmentation pattern with available databases and open literature. Results were expressed as area units normalized to the internal standard, allowing for relative comparisons of the fatty acid abundance between samples.

##### 2.2.4.2 Carotenoids Profile by HPLC-APCI-DAD-MS/MS

The carotenoid profile was tentatively identified in samples obtained via Soxhlet extraction, as this method allowed for efficient solvent removal and yielded a sufficient amount of extract for further analysis. In contrast, extracts obtained using the Me:Eu (1:1) cosolvent system required an energy-intensive evaporation process, which limited material recovery and made it unfeasible to proceed with saponification and subsequent carotenoid analysis.

Saponification was performed following the procedure described by Hoffmann et al.^13^ with slight modifications. Briefly, 2 g of lipid extract were mixed with 3 mL of a methanolic potassium hydroxide solution (10% w/v) and stirred at 56 °C for 20 min. The mixture was then cooled to room temperature for 1 h before the addition of 15 mL of hexane and 30 mL of aqueous sodium sulfate solution (10% w/v). The resulting unsaponifiable fraction was concentrated to approximately 50 mg mL^–1^ for HPLC analysis.

High-performance liquid chromatography (HPLC) analysis of the *B. mori* saponified extracts was performed using an Agilent 1100 series HPLC system (Santa Clara, CA) equipped with a diode array detector (DAD) coupled to an Esquire 2000 ion trap mass spectrometer (Bruker Daltonik GmbH, Bremen, Germany) via an atmospheric pressure chemical ionization (APCI) source. Separation was achieved on a YMC-C30 reversed-phase column (250 × 4.6 mm, 5 μm; YMC Europe, Schermbeck, Germany) with a corresponding C30 guard column (10 × 4 mm, 5 μm). The mobile phase consisted of methanol–MTBE–water (90:7:3, v/v/v) as solvent A and methanol–MTBE (10:90, v/v) as solvent B, with the following gradient elution: 0 min, 100% A; 20 min, 70% A; 35 min, 50% A; 45 min, 20% A; 50–60 min, 0% A; 62 min, re-equilibration to 100% A. The flow rate was set at 0.8 mL/min, and the injection volume was 30 μL. Detection was carried out at 280, 450, and 660 nm, with full UV–vis spectra recorded in the range of 240–770 nm. Mass spectrometric detection was performed in positive ionization mode under the following APCI conditions: capillary voltage, −3.5 kV; drying gas temperature, 350 °C; vaporizer temperature, 400 °C; drying gas flow, 5 L/min; corona current, 4000 nA; nebulizer pressure, 60 psi. Full-scan mass spectra were acquired from *m*/*z* 50 to 1500. Data-dependent MS/MS was performed automatically on the two most intense precursor ions (threshold: 10,000 counts), using a fragmentor amplitude of 1 V. Finally, data processing was achieved through LC ChemStation 3D Software Rev. B04.03 (Agilent Technologies, Santa Clara, CA) and DataAnalysis for the 6300 Series Ion Trap LC/MS Version 4.0 (Bruker Daltonik GmbH, Bremen, Germany).

#### 2.2.5 Biological Activities

##### 2.2.5.1 Antioxidant Capacities

The antioxidant capacity of the lipophilic extracts was determined based on their ability to scavenge free radicals, using the ABTS and DPPH assays, as detailed below.

**DPPH method:** DPPH assay was conducted following the Brand-Williams et al.^19^ protocol. Briefly, 10 μL of each extract (dissolved in ethanol, 10 or 20 mg mL^–1^) was mixed with 290 μL of DPPH ethanolic solution (0.6 μmol L^–1^). Then, the medium was kept for 30 min for reaction at room temperature, and without light, absorbance was measured at 517 nm (BioTek Synergy HT microplate reader, Winooski, Vermont). Trolox was also utilized to construct a reference curve (*R* ^2^ = 0.99). Antioxidant capacity by the DPPH method was expressed in μmol of Trolox equivalent (TE) g^–1^ extract ± standard deviation by triplicate measurements.

**ABTS method:** Antioxidant capacity of lipophilic *B. mori* extracts was also measured using the ABTS method according to the methodology proposed by Re et al.^20^ ABTS+ radical cation was generated by combining a 7 mM ABTS solution with a 2.45 mM potassium persulfate solution without light at room temperature (25 °C) for 16 h. After that, the ABTS+ solution was diluted in distilled water until it reached an absorbance of 0.7 (±0.05) at 734 nm. Finally, 20 μL of diluted extracts (10 or 20 mg mL^–1^) were mixed with 280 μL of ABTS+ solution and incubated in the dark for 30 min, followed by absorbance measurement (BioTek Synergy HT microplate reader, Winooski, Vermont, USA) at 734 nm. Trolox was used as the standard reference curve (*R* ^2^ = 0.99). The results were expressed as μmol of TE g^–1^ extract ± standard deviation from triplicate measurements.

##### 2.2.5.2 Antimicrobial Activity

The antimicrobial activity of *B. mori* extracts was evaluated against *Staphylococcus aureus* (Gram-positive) and *Escherichia coli* (Gram-negative), according to the methodology adapted from Elijah et al.^21^ Bacterial cultures were initially grown in agar medium at 37 °C for 24–48 h. The extracts, diluted in DMSO to a concentration of 10 mg mL^–1^, were applied to agar plates and refrigerated for 30 min to allow for diffusion. Subsequently, 100 μL of *S. aureus* (2.2 × 10^6^ CFU) and *E. coli* (1.2 × 10^7^ CFU) suspensions were then added to the plate containing the test extract, which was properly labeled, and the inoculum was evenly spread over the surface using a Digralsky loop. Erythromycin (0.1 mg mL^–1^) and chloramphenicol (0.1 mg mL^–1^) were used as positive controls, while DMSO and buffered peptone water served as negative controls. Plates were incubated at 37 °C for 24 h. For the interpretation of the results, the presence of a clear inhibition zone around the well indicated a positive result (+), demonstrating the antimicrobial activity of the tested extract. Conversely, the absence of such a zone, with visible bacterial growth covering the entire plate, including the area around the well, was interpreted as a negative result (−), indicating no antimicrobial effect.

### 2.3 Green Assessment by Path2green

The Path2Green software was used to assess the environmental impact and the sustainable performance of the SOX and SFE-NHSolv extraction methods.^22^ The assessment of process sustainability was guided by the 12 principles of green chemistry, which include aspects such as the use of renewable feedstocks (principle 1), transportation efficiency (principle 2), raw material preparation (principle 3), solvent selection (principle 4), scalability (principle 5), purification steps (principle 6), yield optimization (principle 7), postprocessing (principle 8), energy requirements (principle 9), applicability (principle 10), potential for reuse (principle 11), and waste reduction (principle 12). The results were summarized using a pictogram that provided an overall greenness score, in which the highest values indicate the highest sustainability.

### 2.4 Statistical Analysis

The results were expressed as the mean value ± standard deviation. ANOVA followed by post hoc Tukey test was performed when Levene’s test confirmed homogeneity of variances. However, when Levene’s test did not confirm homogeneity of variances for results (possibly because of the small number of observed values, *n* < 4), nonparametric Kruskal–Wallis analysis was performed, followed by Mann–Whitney U test for pairwise comparison whenever applied. All statistical analyses were evaluated using Software Statistica (Statsoft Inc.) under confidence level of 90%.

## 3 Results and Discussion

### 3.1 *B. mori* Pupae Proximal Composition

As a first step in extraction studies, determining the centesimal composition is important for characterizing the sample and guiding the selection of an appropriate method and solvent based on its major constituents. The results for the proximal composition of *B. mori* are shown in Table 1.

**Table 1:** Proximal composition (%) of *B. mori* subjected to blanching followed by freeze-drying (B+FD) and only to freeze-drying (FD) as pretreatments^a^

	***B. mori*** **pretreatment**	
**component** (%)	**FD**	**B+FD**
**moisture**	7.56^a^ ± 0.20	2.18^b^ ± 0.08
**protein**	36.62^a^ ± 1.00	36.25^a^ ± 0.41
**lipids**	35.05^a^ ± 0.85	37.77^b^ ± 0.21
**carbohydrates**	17.81^a^ ± 1.52	20.05^a^ ± 0.77
**ashes**	4.42^a^ ± 0.08	3.69^b^ ± 0.12

a Different superscript letters (a, b) in the same row indicate significant differences between treatments (*P* ≤ 0.1).

The centesimal composition of *B. mori* (Table 1) shows a predominance of lipids and proteins, in agreement with values reported in the literature. The protein content observed in the present study (∼36%) was lower than values reported by Akande et al.,^23^ who reported 47.17% for protein extracted from blanched *B. mori* pupae. In another study presented by the same authors,^24^ a higher protein content (60.7%) was found, where freshly harvested *B. mori* pupae were first stifled at 93 °C for 1 h, then removed from their cocoons and subsequently dried in a hot air oven at 40 °C. In this study, *B. mori* larvae were blanched with the cocoon intact, which likely acted as a physical barrier that minimized the leaching of soluble proteins into the blanching medium, thereby preserving protein content. In contrast, in this study, blanching was performed without the cocoon, potentially increasing the direct exposure of larval tissues to hot water and promoting protein solubilization and loss. The differences in the procedures may help explain the lower protein content observed.

The lipid fraction in both B+FD and FD samples (37.77 and 35.05%, respectively) was closer to the values reported for blanched *B. mori* (32.16%)^24^ and higher than those obtained by only dried insects (23.5%).^23^ Carbohydrate levels were lower (17.81–20.05%) than lipids and proteins, highlighting the predominance of these macronutrients in *B. mori*. However, in biorefinery studies, this amount is significant, as carbohydrates represent a valuable fraction that can be recovered. Finally, the ash content was greater in the present samples (3.69–4.43%) than previous results indicated in the literature (0.9–2.12%),^23,24^ demonstrating a higher mineral residue.

The differences observed between the present study and values reported in the literature may be explained by factors such as silkworm strain, pupal developmental stage, insect diet, environmental conditions, and the analytical methods employed.^25^ These variables can influence the proximate composition, making it difficult to achieve consistent nutritional assessments across studies. This complexity underscores the importance of standardized methodologies and carefully controlled processing strategies in maintaining the nutritional quality of edible insects throughout the entire production chain.

### 3.2 Extraction Yield Efficiency

Since lipids are the major components of *B. mori* pupae (together with proteins) and have not been widely studied, in the present work, we focused on developing a sustainable extraction process to isolate *B. mori* lipid fraction and to evaluate their composition and potential bioactivities. The extraction process consisted of supercritical fluid extraction (SFE) using carbon dioxide plus an emergent hydrophobic cosolvent (Me:Eu, 1:1 w/w) that was tested in order to favor the recovery of the lipid fraction. Conditions were selected based on a recent work of our research group using *G. mellonella* larvae as a raw material. Moreover, the performance of this green approach was compared with that of a conventional Soxhlet extraction using hexane, as illustrated in Figure 1.

As depicted in Figure 2, the extraction yield varied significantly among the evaluated methods, with SFE combined with natural-based solvents and blanching+freeze-drying (SFE+NHSolv B+FD) presenting the highest value (55.98%). This result reflects the superior efficiency of SFE in combination with green cosolvents such as Me:Eu, which enhance solubility and selectivity for lipid-soluble and mildly polar compounds. It is also important to point out that, considering that the oil content of the raw material is 37.77% (Table 1), the synergic effect of natural cosolvents (SFE+NHSolv) and the pretreatment (B+FD) likely enhanced not only lipid solubility but also promoted the extraction of additional polar compounds, such as partially hydrolyzed proteins and sugars. As a result, the purity of the extract was lower than that obtained with freeze-drying (FD) treatment alone. Therefore, in applications where higher purity or selective lipid recovery is required, FD pretreatment may be a more suitable option.

**Figure 2:**
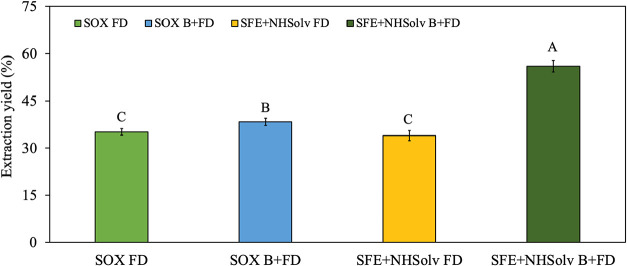
Extraction yield of *B. mori* extracts obtained using Soxhlet (SOX) and supercritical fluid extraction with natural-based solvents (SFE-NHSolv) considering freeze-drying (FD) and blanching+freeze-drying (B+FD) as pretreatments. Different superscript uppercase or lowercase letters indicate significant differences between treatments (*P* ≤ 0.1).

The SOX method, whether applied to freeze-dried (SOX FD) or blanched and freeze-dried (SOX B+FD) samples, achieved high total lipid recoveries (35.1 and 38.3%, respectively), although no significant differences were observed when compared to SFE+NHSolv considering FD as pretreatment, and higher values were achieved for the SFE+NHSolv using B+FD. Moreover, Soxhlet involves prolonged heating, lacks selectivity, and relies on hexane, a solvent associated with environmental and safety concerns.

Notably, the effect of sample pretreatment was evident when comparing FD and B+FD conditions: blanching before freeze-drying resulted in a slight increase in yield for both extraction methods. This improvement may be attributed, in part, to the blanching step, which promotes softening and partial disruption of the tissue structure, denaturing structural proteins in both the exoskeleton and internal compartments, increasing matrix porosity, and enhancing solvent penetration and diffusion during extraction.^5,26^ A recent study by Lee et al.^5^ confirmed that blanching, compared to other thermal treatments, significantly reduced bulk density and increased rehydration capacity, indicating structural loosening and increased porosity in *Hermetia illucens* larvae, which favored mass transfer. This behavior partially explains the enhanced solvent diffusion and compound accessibility observed in the present study, contributing to the increased extraction yield.

Furthermore, the combination of pretreatment (blanching and freeze-drying) with supercritical CO_2_ extraction at high pressures produced a synergistic effect that amplified the overall extraction performance. Under supercritical conditions, carbon dioxide exhibits gas-like diffusivity and liquid-like solvating power, allowing for deep penetration into the porous matrix and the effective solubilization of target compounds.^14^ The incorporation of natural-based cosolvents, menthol, and eucalyptol further enhanced the system by broadening its polarity range. Due to their amphiphilic properties, these solvents enhance the solubility of both nonpolar and moderately polar compounds, thereby increasing extraction efficiency and selectivity.^27^ Overall, the improved performance observed with the SFE+NHSolv B+FD method can be attributed to the combined effects of matrix modification, the use of a tailored solvent system (Me:Eu), and the selected extraction parameters.

Compared to the study by Srinivas et al.,^28^ which reported an extraction efficiency of 30.10% under optimized conditions (45 °C, 203 bar, 145 min, and CO_2_ flow rate of 24 g min^–1^), the present study achieved substantially higher yields (35–56%) using a slightly higher temperature (60 °C), similar pressure (200 bar), significantly shorter extraction time (90 min), and lower CO_2_ flow rate. Another study by Nam et al.^29^ reported the extraction of *Tenebrio molitor* oil using SFE (400 bar, 55 °C, and a 3-h extraction time), yielding a lower yield of 25.43%. Although derived from different insect species, this comparison highlights the efficiency of the present method for *B. mori*, which achieved higher yields under milder conditions. Notably, the incorporation of a natural hydrophobic cosolvent in the present study contributed to enhanced extraction efficiency by improving solubility and facilitating the recovery of the target compounds.

Interestingly, a similar trend was observed in the study by dos Santos et al.^17^ in which a yield of 58% was reported for *G. mellonella* larvae using the same extraction conditions applied in the present study (SFE with 15% Me:Ci as cosolvent, 200 bar, 60 °C, and 4 mL min^–1^). Furthermore, SFE-NHSolv also demonstrated higher efficiency compared to Soxhlet extraction (50.4%), reproducing the behavior observed in our findings. These results suggest that the green technique proposed in the present work may be reproducible for insect matrices with comparable proximate compositions, especially in terms of lipid and protein content, which can significantly influence solvent accessibility and extraction performance.

Another approach that has been explored for *B. mori* oil extraction is the Aqueous saline process. In a study by Tangsanthatkun et al.^30^ this method yielded a significantly lower oil content of 3.32% under specific saline and stirring conditions (1.7% w/v saline solution, a liquid-to-solid ratio of 3.3 mL g^–1^, and 119 min of stirring at 100 rpm). In contrast, the present study achieved substantially higher yields (35–56%), underscoring the potential of high-pressure extraction for improving the oil yield.

Overall, SFE-NHSolv represents a cleaner and more sustainable alternative for lipid extraction from *B. mori*, reducing environmental impact while maintaining high performance. In addition to the efficient oil recovery, the resulting defatted pellet may serve as a valuable biomass for the recovery of proteins and other compounds of interest. Notably, the high pressure applied during extraction can promote cell disruption, thereby enhancing the accessibility of intracellular components and the efficiency of subsequent steps within an integrated biorefinery approach.

### 3.3 Extract Characterization

#### 3.3.1 Fatty Acids Profile

Fatty acid analyses were conducted to compare the lipid profiles obtained from SFE-NHSolv and SOX extraction methods, aiming to understand the effect of the technique and the pretreatment on the composition of the extracted fatty acids. This comparison is particularly relevant given that the fatty acid profile is a key quality parameter for potential applications in food, cosmetic, and pharmaceutical industries. The resulting profiles of *B. mori* extracts are presented in Figure 3, while the chromatograms are depicted in Figure S1 (Supporting Information). The identification of fatty acids by GC-MS of SFE-NHSolv and SOX extracts and their detailed statistical analysis are also presented in the Supporting Information (Tables S1 and S2).

**Figure 3:**
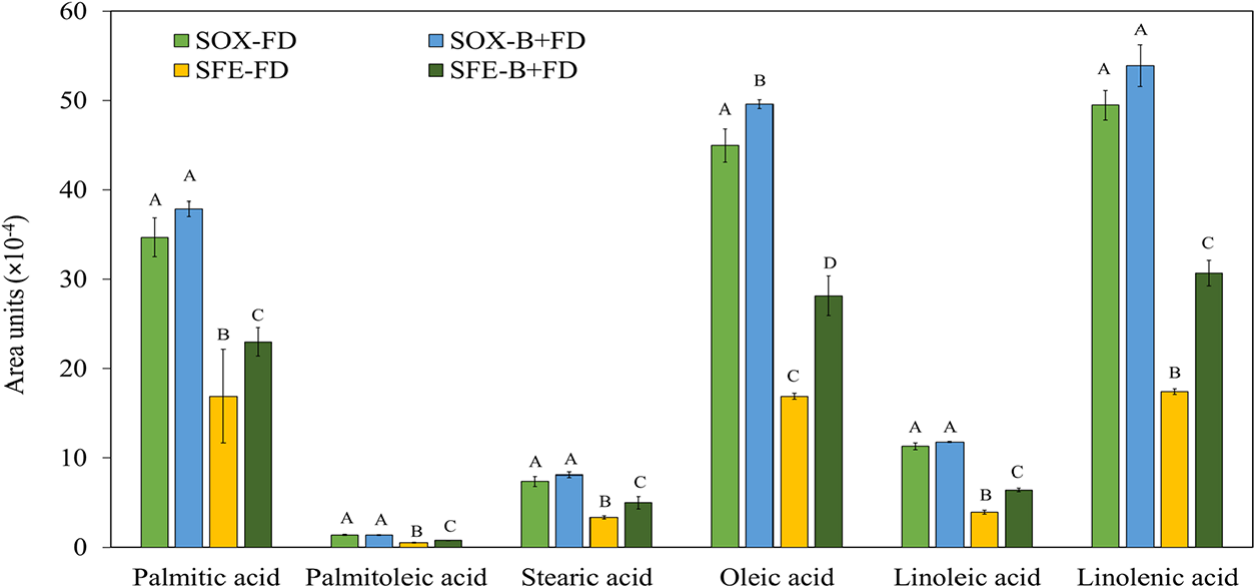
Fatty acid profile of lipophilic *B. mori* pupae extracts obtained by Soxhlet (SOX) and supercritical fluid extraction with natural-based solvents (SFE-NHSolv) considering freeze-drying (FD) and blanching+freeze-drying (B+FD) as pretreatments. Different letters within the same fatty acid indicate statistically significant differences between extraction treatments (*P* ≤ 0.1).

As can be observed, main fatty acids identified in the different extracts were α-linolenic, oleic, and palmitic acids; these results align with recent studies on *B. mori* pupae characterization using GC–MS; in this sense, Yeruva et al.^31^ reported that lipids represent ∼30% of pupal dry weight, prominently featuring also α-linolenic, oleic, and palmitic acids through GC–MS analysis. Additionally, a comprehensive review by Tassoni et al.^25^ consistently reported a high content of α-linolenic acid, along with a broad profile of other essential fatty acids, in *B. mori* oil. These findings corroborate the high levels observed in this study and emphasize that *B. mori* pupae are a consistent and rich source of essential fatty acids for food, cosmetic, and pharmaceutical fields.

As previously described (Section 2.2.4.2), heptadecanoic acid (C17:0) was added in a fixed amount to each sample as an internal standard, allowing for a reliable comparison of fatty acid peak areas across different extraction methods. This approach enables the assessment of how each technique influences the relative abundance of individual fatty acids. The comparative analysis of extraction methods shows that SOX-B+FD yields the highest relative abundance of key fatty acids, particularly linolenic acid (53.93 × 10^4^), oleic acid (49.58 × 10^4^), and palmitic acid (38.96 × 10^4^). Compared to the same pretreatment, when using SFE-NHSolv (SFE-B+FD), the same fatty acids achieved relatively lowest abundance, highlighting the substantial differences between the extraction methods (*p*-values of 0.03, 0.03, and 0.03, respectively, Table S2).

By comparing the different pretreatments, it can be clearly seen that the effect produced by blanching, mainly when using SFE as the extraction process. This behavior may be explained by the biochemical and structural effects induced by blanching. As already mentioned, the treatment promotes the partial disruption of cellular structures and denaturation of membrane proteins, increasing membrane permeability. It also inactivates endogenous enzymes such as lipases and lipoxygenases, which could otherwise degrade or oxidize unsaturated fatty acids during or after freeze-drying.^6,32,33^ Although Soxhlet extraction promotes lipid solubilization due to prolonged exposure to hot solvent, the blanching pretreatment proved to be more effective in improving the recovery of specific lipid fractions, particularly palmitic (C16:0), oleic (C18:1), and linolenic (C18:3) acids. In contrast, minor fatty acids such as palmitoleic (C16:1), stearic (C18:0), and linoleic (C18:2), which are present in lower amounts and likely less affected by blanching-induced structural changes, did not exhibit significant differences between treatments. The opposite trend was observed for SFE, where samples subjected to blanching followed by freeze-drying exhibited significantly higher chromatographic peak areas for all fatty acids in comparison to the samples that were only freeze-dried. These findings can be attributed to the combined effects of high-pressure CO_2_ penetration^34^ and the structural modifications induced by blanching, which enhance matrix permeability and facilitate solvent–lipid interaction.^32^ Again, the synergistic effect between tissue modification and enzyme inactivation may explain the consistently higher peak areas of both major (palmitic, oleic, and linolenic) and minor (palmitoleic, stearic, and linoleic) fatty acids when blanching was applied prior to freeze-drying.

Further, as presented in Section 3.2, a comparison between the Soxhlet and SFE methods revealed that the overall lipid yield was similar. Nevertheless, despite its high efficiency in extracting a wide range of lipid classes, GC-MS analysis revealed consistently higher peak areas for individual fatty acids in SOX samples, suggesting a greater relative abundance of these compounds in those extracts. The lower GC-detected fatty acid values likely reflect differences in lipid class composition, as SFE may favor the extraction of nonsaponifiable lipids or complex lipid molecules, such as sterols, partial glycerides, phospholipids, and wax esters^35^ that are not fully converted into fatty acid methyl esters (FAMEs) during derivatization.

Recent studies on insect lipid extraction support these findings. For instance, Fornari et al.^34^ demonstrated that SFE extraction applied to *H. illucens* larvae resulted in high total lipid recovery while also enabling the extraction of minor compounds such as squalene and phytosterols. Similarly, Franco et al.^36^ reviewed how extraction methods applied to different kinds of insects influence the lipid profile, showing that although fatty acid profiles remained broadly consistent, notable differences occurred in lipid subclasses (e.g., wax esters, glycerides, and sterols).

In contrast, Hurtado-Ribeira et al.^37^ evaluated the effects of different slaughtering, drying, and defatting methods on the lipid composition of *H. illucens* larvae. The authors observed that despite variations in processing, the overall fatty acid distribution, particularly in terms of the fatty acid profile, remained stable. Minor lipid constituents, such as phytosterols and squalene, were also identified. These findings differ from those of the present study, in which significant differences in individual fatty acid contents were observed between Soxhlet and SFE-NHSolv extracts of *B. mori* pupae. A contributing factor is the use of a natural-based hydrophobic solvent (Me:Eu 1:1) as a cosolvent in the SFE system, which may have altered extraction selectivity by enhancing the solubility of other lipid classes. These findings highlight the promising effects of both extraction techniques and solvent composition, since they can significantly influence the resulting lipid profile of insect extracts.

Compared with other larvae reported in the literature, *B. mori* shows clear advantages in its fatty acid profile, especially when compared to *G. mellonella*,^17^ which was evaluated under similar extraction conditions using natural-based solvents. While *G. mellonella* contained oleic (39%) and linoleic (12%) acids, linolenic acid was not detected. In contrast, *B. mori* presented a higher proportion of unsaturated fatty acids, including a notable amount of linolenic acid. This advantage enhances its nutritional value and positions *B. mori* as a more promising edible insect source, particularly due to the presence of linolenic acid, which is associated with health-promoting effects.

The fatty acid composition of *B. mori* shows partial similarity to that of common edible oils, such as olive,^38^ particularly in the relative proportions of oleic acid (70–74%) and linoleic acid (10–14%). However, it differs in terms of palmitic acid (C16:0) content, which ranges from 9 to 11% in olive oil and is generally higher in *B. mori*. Given its favorable profile, rich in monounsaturated and polyunsaturated fatty acids, *B. mori* oil could represent a promising alternative for formulating lipid blends intended for interesterification processes. Such blends may be suitable for use in bakery products, especially when combined with other insect-derived oils to achieve the desired functional and nutritional properties.

#### 3.3.2 Carotenoid Profile

The yellowish color presented by *B. mori* extracts suggested the presence of carotenoids, known for contributing yellow to orange pigments in lipid-rich matrices. Based on this observation and previous reports of carotenoids in insect oils, the chromatographic profile and a tentative identification of the detected compounds in saponified SOX FD and SOX B+FD extracts are presented in Figure 4 and Table 2, respectively.

**Figure 4:**
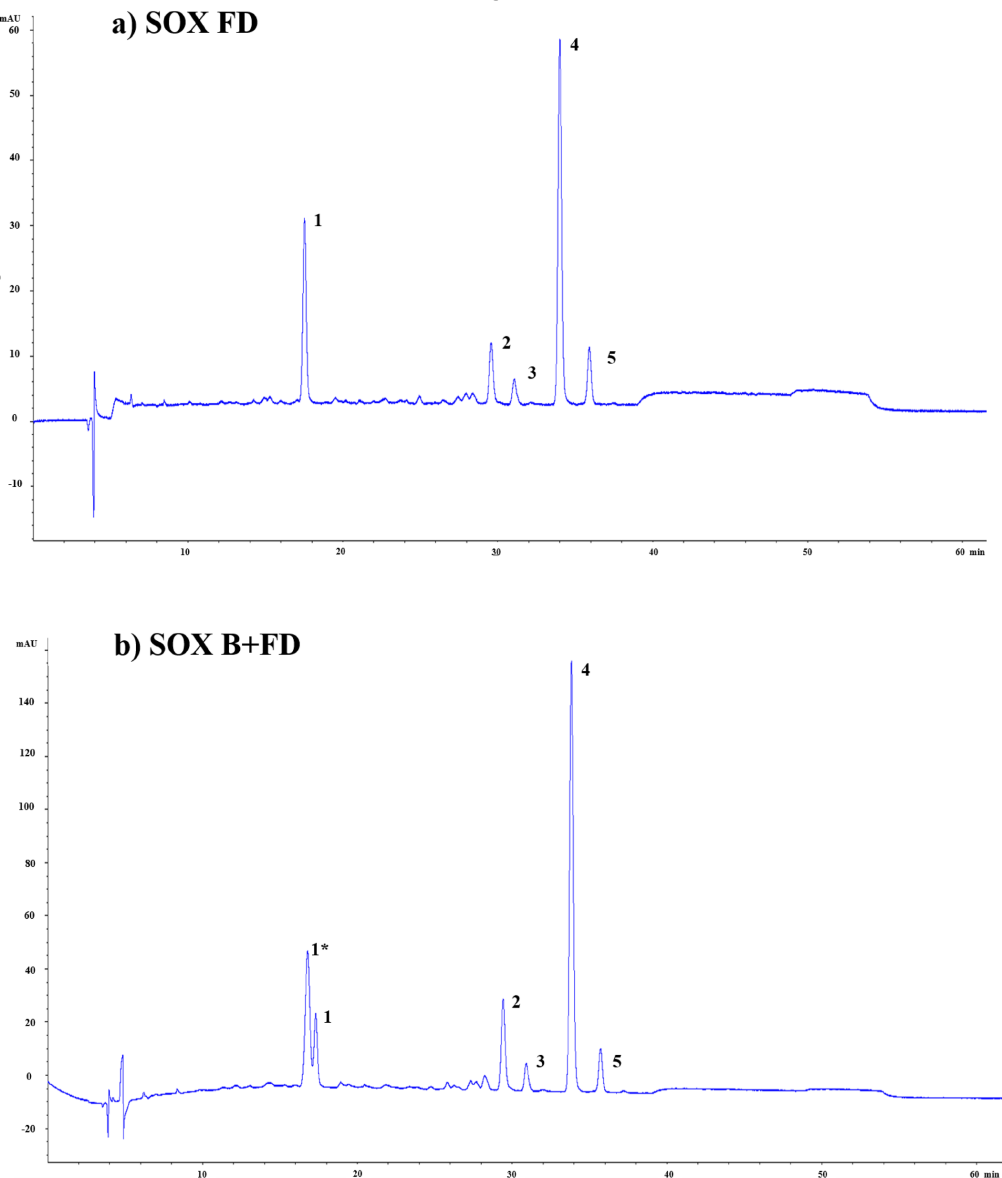
HPLC-DAD chromatograms obtained at 450 nm for Soxhlet saponified extracts from *B. mori* pupae pretreated by a) freeze-drying (SOX FD) or b) blanching + freeze-drying (SOX B+FD).

**Table 2:** Tentative Identification of Carotenoids in *B. mori* Pupae Pretreated by Blanching + Freeze-Drying (SOX B+FD) or Freeze-Drying (SOX FD) by HPLC-DAD-APCI-MS/MS (Peak # Assignments Shown in Figure 3)

peak #	RT (min)	UV–vis max (nm)^a^	%III/II^b^	main fragments (*m*/*z*)	tentative identification	reference(s)	notes
1*	16.7	418s, 443, 472	75	551.4 (precursor type [M+H–H_2_O]^+^), 533.5, 411.3, 359.2, 495.3, 429.3, 459.3	lutein	Soares et al.^42^ and Breemen et al.^39^	fragmentation shows water loss. parent ion [M + H]^+^ not detected, common for xanthophylls in APCI-ion trap MS
**1**	17.3	420s, 445, 473	71	551.5 (precursor type [M+H–H_2_O]^+^), 429.3, 533.3, 261.2, 197.0, 173.0, 459.3	zeaxanthin (lutein derivative)	Pavelková et al.^43^ and Rivera et al.^44^	similar fragmentation to lutein; elution order on C30 column supports ID
**2**	29.5	445, 470	25	not fragmented	unknown carotenoid		broad range of carotenoids with same UV–vis spectral information; no MS^2^ data obtained
**3**	31.0	445, 475	50	not fragmented	unknown carotenoid		likely present in very low concentration; no MS^2^ data obtained
**4**	34.0	425s, 450, 480	61	537.6 (precursor type [M + H]^+^), 399.3, 445.6, 177.1, 361.2	β-carotene	Van Breemen et al.^39^	identification confirmed by standard
**5**	35.9	420s, 445, 475	62	578.5 (precursor, unknown type), 265.1, 239.1, 313.2, 247.1, 321.3	β-carotene derivative		UV–vis spectrum similar to β-carotene; possible oxidation product. fucoxanthin suggested^45^ by UV–vis, but unlikely due to its algal origin (not a *B. mori* diet-related compound)

a Maximum wavelength underlined; s = shoulder.

b Spectral fine structure represented as the % ratio of the height of the longest-wavelength absorption peak (III) and that of the middle absorption peak (II).

Among the carotenoids tentatively identified in *B. mori* pupae extracts (Table 2), lutein (compound 1*) and zeaxanthin (a lutein derivative, compound 2) were detected, with lutein observed exclusively in the blanched+freeze-dried (SOX B+FD) samples. The fragmentation patterns observed for lutein and zeaxanthin are consistent with previously reported data for xanthophylls analyzed by APCI-MS/MS, including characteristic water loss and the absence of intact [M + H]^+^ ions.^39^ The presence of lutein in the SOX B+FD sample suggests that the blanching step effectively inactivated endogenous enzymes that could promote lutein oxidation, thus preserving this compound, which was not observed in the SOX FD sample, likely due to the absence of high temperature (from blanching step).^40,41^ This highlights the importance of enzyme inactivation prior to extraction to prevent the loss of lutein and other compounds that may be susceptible to enzymatic oxidation processes.

While studies directly investigating lutein preservation in insects are limited, similar enzymatic degradation mechanisms have been well documented in vegetables, where blanching prior to drying is essential to preserve lutein and other carotenoids.^46,47^ By analogy, it is reasonable to expect that blanching also plays a crucial role in preserving lutein and other bioactive compounds in edible insects. Conversely, β-carotene (compound 4) was identified in both extracts. The persistence of β-carotene in thermally treated samples reinforces its known resistance to degradation under moderate heat, unlike that of lutein, which was not detected after thermal processing.

Similar carotenoids have been reported in other edible insects, such as *T. molitor*,^48^ *Locusta migratoria*,^49^ and *Halyomorpha halys*.^50^ These findings demonstrate that edible insects can serve as relevant sources of carotenoids, such as lutein, β-carotene, and zeaxanthin, but also emphasize that the specific carotenoid profile varies considerably depending on the insect species, developmental stage, and especially the composition of their diet.

Interestingly, a distinct peak at 22.06 min was detected at 280 nm and tentatively identified as a carotenoid based on its UV spectral characteristics (Figure S2, Supporting Information). However, MS^2^ fragmentation data could not be obtained for this compound. In this chromatographic region, colorless carotenoids such as phytoene and phytofluene are commonly observed due to their absorption in the near-UV range. Nevertheless, their known absorption maxima, 285 nm for phytoene, and 331, 347, and 365 nm for phytofluene^53^ differ markedly from the spectral profile observed in this study. This discrepancy suggests the possible presence of lutein or zeaxanthin derivatives, such as apocarotenoids, which are known to absorb around 280 nm.^51^ These compounds may originate from the mulberry leaf diet of the larvae or may be produced through their own metabolic pathways. The detection of carotenoid-like peaks lacking MS/MS confirmation highlights both the limitations of nonquantitative methods and the chemical complexity of insect pigments.

### 3.4 Biological Activity Assessment

The antioxidant capacity of the extracts was evaluated to assess the potential of the lipid fractions to neutralize free radicals and enhance their functional properties, particularly in food, nutraceutical, and cosmetic applications. Although the lipophilic ORAC assay was initially tested in this study as it is considered suitable for assessing the antioxidant activity of lipophilic fractions, the high complexity of the extract (comprising multiple nonpolar constituents such as waxes, lipids, and other hydrophobic compounds) caused turbidity and phase instability in the reaction medium upon addition. This interfered with fluorescence measurements and resulted in poor reproducibility. Therefore, DPPH and ABTS assays were selected to evaluate the antioxidant activity of the extracts, as they are widely applied and suitable for assessing a broad range of antioxidant compounds in complex lipid-based systems.^52,53^

The results of the DPPH and ABTS assays are listed in Figure 5.

**Figure 5:**
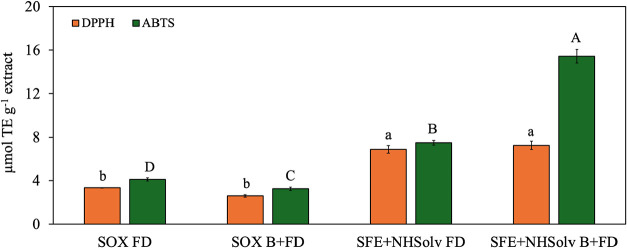
Antioxidant capacity of *B. mori* extracts obtained using Soxhlet (SOX) and supercritical fluid extraction with natural-based solvents (SFE-NHSolv) considering freeze-drying (FD) and blanching+freeze-drying (B+FD) as pretreatments. Different superscript uppercase or lowercase letters indicate significant differences between treatments (*P* < 0.1).

In general, extracts obtained using SFE with a natural hydrophobic solvent (SFE+NHSolv) exhibited a higher antioxidant capacity compared to Soxhlet-extracted samples. The SFE-NHSolv B+FD treatment exhibited the highest radical scavenging capacity, with values of 7.25 μmol TE g^–1^ extract for DPPH and 15.45 μmol TE g^–1^ extract for ABTS. In contrast, Soxhlet extracts (SOX FD and SOX B+FD) demonstrated lower efficiency in reducing free radicals, with the highest ABTS value observed in the SOX B+FD (3.25 μmol TE g^–1^) extract. The differences in the antioxidant capacity can be attributed, in part, to the extraction method and solvent characteristics: supercritical CO_2_ combined with a hydrophobic cosolvent such as Me:Eu (1:1) allows for better recovery of a broader range of antioxidant compounds, including medium polar and amphiphilic molecules that may not be efficiently extracted by hexane, corroborating with the yield (Section 3.2) and fatty acids (Section 3.3) results.

The antioxidant capacity observed in the SOX extracts may be partly attributed to the identified carotenoids (Section 3.3.2). Although carotenoid analysis was not performed for the SFE-derived extracts, these compounds may also be present, given the lipophilic nature of the insect matrix. Moreover, the higher antioxidant capacity observed in SFE samples suggests that, in addition to carotenoids, other compounds, such as sterols, partial glycerides, phospholipids, wax esters, and polyphenols, as previously discussed for fatty acids (Section 3.3.1), may contribute synergistically to the whole phytochemical profiling in SFE extracts and therefore play a role in the overall antioxidant performance.

Results for antioxidant capacities for *B. mori* pupae vary significantly across studies, influenced by factors such as the extraction method, solvent type, and biological conditions, including diet, strain, and developmental stage, as previously mentioned. However, a clear pattern emerges in the literature regarding *B. mori* extractions that aligns with the present findings: ABTS values tend to exceed those of DPPH. For instance, Anuduang et al.^54^ reported DPPH and ABTS values of 22.43 and 35.37 μmol TE g^–1^, respectively, for ethanol extracts obtained from blanched and dried pupae. Similarly, Wang et al.^55^ evaluated aqueous extracts obtained by subcritical water extraction (200 °C), and the trend remained consistent, with ABTS showing greater antioxidant capacity (IC_50_: 1.5 mg mL^–1^) than DPPH (IC_50_: 10 mg mL^–1^). While it is not methodologically appropriate to compare aqueous extracts with the lipid-based fractions used in this study, these results collectively reinforce the broader sensitivity of the ABTS assay to a wider spectrum of antioxidant compounds across various extraction approaches. This behavior can be attributed to the fact that ABTS detects a broader spectrum of antioxidant compounds, including both hydrophilic and lipophilic ones. In contrast, DPPH is more limited to nonpolar molecules.^53^ Although the absolute values differ, the trend observed here reinforces the complementarity of both assays in assessing the antioxidant potential of complex extracts.

In summary, the results suggest that the use of SFE combined with a natural cosolvent and blanching pretreatment prior to freeze-drying plays a significant role in enhancing the antioxidant capacity of the recovered lipid extracts for potential applications in food preservation, cosmetic formulations, and nutraceuticals, where oxidative stability and bioactivity are critical attributes.

### 3.5 Antibacterial Properties

The antimicrobial activity of extracts SOX and SFE-NHSolv was assessed by using the agar well diffusion method. The extracts were evaluated against *S. aureus* and *E. coli*, selected as clinically relevant representatives of Gram-positive and Gram-negative bacteria, respectively. Results are presented in Supporting Information at Figure S3.

As can be seen, none of the tested *B. mori* extracts produced inhibition halos against either bacterial strain, indicating a lack of detectable antimicrobial activity at an extract concentration of 10 mg mL^–1^. These findings are consistent with the results reported by Suman et al.^3^ in which *B. mori* extracts (from Soxhlet method using hexane as solvent) did not exhibit bactericidal activity against any of the tested strains, including *Bacillus cereus*, *Clostridium sporogenes*, *S. aureus*, *E. coli*, *Enterobacter cloacae*, and *Salmonella typhi*. In contrast, *B. mori* pupal oil obtained through mechanical pressing showed moderate antimicrobial activity against *Bacillus subtilis* and *S. aureus* (at 1:1 oil:DMSO).^3^ It is worth noting that the exact concentration used by the authors’ study could not be determined since the authors expressed the oil dilution as a volume-to-volume ratio, which prevents a direct comparison with the concentrations applied in the present work. Despite the possible bioactivity and antioxidant properties (Sections 3.3.2 and 3.4), antimicrobial effectiveness of *B. mori* extracts seems to depend on factors such as extraction method, extract concentration, and the specific microorganisms involved.

### 3.6 Green Assessment by Path2green

The sustainable performance of the SOX and SFE-NHSolv extraction processes was assessed using the Path2Green software,^22^ which is briefly discussed below.

Principle 1 relates to the use of renewable feedstocks. In this study, *B. mori* insects are considered byproducts of sericulture, which aligns with the principle of utilizing biomass that does not compete with food sources or require additional cultivation (score +1). The environmental impact associated with transportation is considered in principle 2. A hypothetical local transport scenario was assumed for both SOX and SFE, involving moderate distances (50 km), efficient vehicles, and eco-friendly packaging. As an important role in extraction processes, principle 3 relates to the pretreatment of raw materials, which should enhance extraction efficiency with minimal environmental impact. The physical methods used in this study (blanching, freeze-drying, and grinding) scored −0.20. After pretreatment, appropriate solvents were selected (Principle 4). The use of hexane in the Soxhlet method was against green chemistry principles (score −1). In contrast, the SFE method utilized CO_2_, aligning well with the sustainability criteria (score of +1). Principle 5 assesses scalability, in which SFE can operate in a semicontinuous mode, allowing for uninterrupted and flexible processing (score 0.5). In contrast, Soxhlet operates in batch mode (score −1). The following principle addresses purification needs (Principle 6). While purification can be essential for high-purity applications, SFE-NHSolv extracts may be ready-to-use extracts since cosolvent concentrations comply with regulatory limits^8,15^ eliminating the need for further purification. This approach makes the SFE particularly advantageous for streamlined processing.

It is important to note that in ready-to-use extracts, regulatory compliance, allergenicity, and compound stability must be carefully considered, as these factors directly affect product safety, efficacy, and quality.

For example, regarding cytotoxicity, recent studies have demonstrated that 1,8-cineole (eucalyptol) exhibits minimal cytotoxicity toward normal human cells. For instance, Rodenak-Kladniew et al.^56^ reported an IC_50_ > 10 mM in WI-38 normal lung fibroblasts, compared to a lower IC_50_ of 5.84 mM in A549 cancer cells. Similarly, Naksawat et al.^57^ reported strong cytotoxic effects of menthol on NB4 and Molt-4 leukemia cell lines (IC_50_ = 250–300 μg mL^–1^), while exhibiting minimal impact on normal peripheral blood mononuclear cells (PBMCs), which maintained >90% viability at 300 μg mL^–1^.

Regarding the regulatory status, menthol and 1,8-cineole, the solvents present in SFE-NHSolv extracts, have well-established regulatory approval and favorable safety profiles. Both compounds are Generally Recognized as Safe (GRAS) by the U.S. FDA for use in foods and flavorings.^17^ They are also permitted as ingredients in cosmetics and nutraceutical formulations under EU Regulation (EC) No 1223/2009. However, they are not listed among the EU’s mandatory fragrance allergens (Annex III, EC 1223/2009).^58^ However, formal allergenic and cytotoxicity studies are recommended for further studies, particularly for topical applications, to ensure that concentrations in the extracts remain well below levels that could induce adverse reactions.

Another important consideration is that the stability of volatile compounds, such as menthol and 1,8-cineole, can be affected by temperature, light, and oxygen during storage. A study by Ganosi et al.^59^ demonstrated good storage stability for both compounds over a six-month period. In *Origanum vulgare* L. essential oil, the 1,8-cineole content decreased from 1.37 to 0.71% after six months of storage in sealed clear glass tubes at 23 °C in the dark. In contrast, menthol from *Mentha spicata* L. declined from 3.01% at week 10 to 0.98% after six months. These findings indicate that under controlled storage conditions, the chemical integrity and bioactive properties of menthol and 1,8-cineole in ready-to-use extracts can be effectively maintained.

In summary, it is possible to suggest that ready-to-use extracts containing menthol and 1,8-cineole obtained in the present study can be safely applied in food, cosmetic, and nutraceutical products, provided that regulatory requirements are met, cytotoxicity and allergenicity are properly assessed, and storage conditions are optimized. Addressing these factors ensures the preservation of bioactive properties, product safety, and long-term stability.

Soxhlet extracts, on the contrary, often require further purification due to the use of nonselective solvents like hexane. Therefore, a score of +1 and −1 was assigned to SFE-NHSolv and SOX, respectively.

Principle 7 evaluates biomass utilization and extraction yield. In both methods, the residual biomass could still contain valuable compounds, particularly peptides, which could be recovered in subsequent steps. Therefore, both methods scored −1. Principle 8, different from Principle 6, focuses on post-treatment steps related to safety and extract readiness for use. SFE produces extracts that are essentially ready for direct application, as CO_2_ and natural cosolvents are safe, requiring minimal or no post-treatment (score +1). On the other hand, Soxhlet extraction involves hexane, a toxic solvent that demands more complex post-treatment steps to remove residual solvent and ensure extract safety (score −1).

Principle 9 considers the type and efficiency of energy used. Soxhlet requires prolonged heating at higher temperatures (>70 °C), resulting in high thermal energy consumption, while SFE requires high energy inputs for CO_2_ compression and pumping. Therefore, both methods were assigned a score of −1 due to their use of nonrenewable energy, although it is worth mentioning that SFE operates at lower temperatures (40–60 °C) and is significantly faster.

Principles 10 and 11 highlight the safety and broad applicability of the extracts and the recovery and reuse of solvents, respectively. Due to their antioxidant and bioactive properties, lipid fractions from *B. mori* show potential across multiple sectors, such as functional ingredients in food or cosmetics. Additionally, residues may serve as natural additives or biofertilizers, contributing to sustainable agricultural practices.^4,60,61^ Regarding solvent recovery, in SFE-NHSolv, CO_2_ is recovered in a closed-loop system, while the natural cosolvent can be directly incorporated into the final product as a ready-to-use extract. In contrast, Soxhlet uses hexane in an open system, resulting in greater solvent losses and a higher environmental impact. Thus, these characteristics classified both methods with score 0.66 for Principle 10, and for SFE-NHSolv and Soxhlet, the scores were +1 and −1 for Principle 11, respectively.

Finally, Principle 12 highlights the importance of minimizing waste for sustainable extraction (when no process integration is applied). SFE-NHSolv generates less waste due to its higher extraction efficiency, resulting in 44% of generated waste (input score in the software). In contrast, Soxhlet exhibits a lower efficiency, resulting in greater waste generation and an input score of 65%. In conclusion, the sustainability scores for SOX and SFE-NHSolv were −0.328 and 0.472 (Figure 6), respectively, indicating that the SFE-NHSolv method is considerably more sustainable than the conventional Soxhlet extraction.

**Figure 6:**
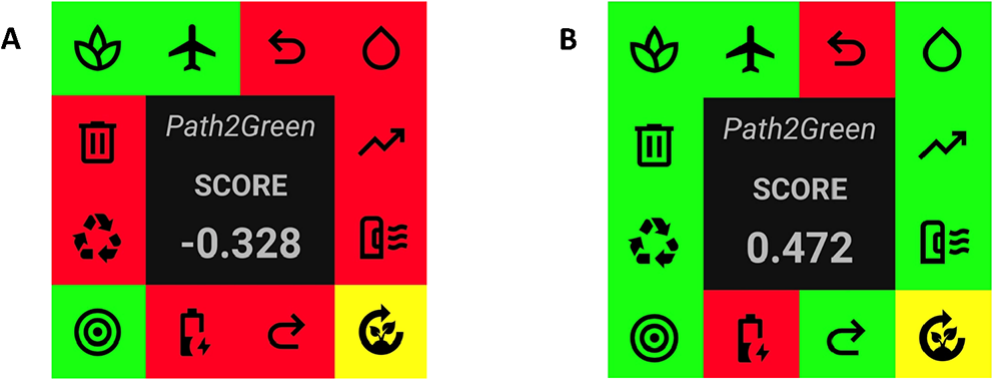
Comparative environmental impact assessment of (A) SOX and (B) SFE-NHSolv. Credit: *Path2Green* software.

## 4 Conclusion

As a byproduct of the silk industry, silkworm (*B. mori*) pupae offer great potential for valorization as a rich source of lipids. This study successfully demonstrated that SFE-NHSolv, combined with blanching and freeze-drying pretreatments, is a more efficient and sustainable alternative to conventional Soxhlet extraction for recovering lipophilic compounds from *B. mori* pupae. The optimized SFE-NHSolv B+FD process achieved the highest extraction yield of 55.98%, significantly outperforming the SOX-B+FD yield of 38.3%. Besides that, SFE-NHSolv B+FD exhibited enhanced antioxidant capacity, with significant results for ABTS (15.44 μmol TE g^–1^) and DPPH (7.23 μmol TE g^–1^). GC-MS analysis revealed that the lipid extracts from *B. mori* pupae are primarily composed of linolenic, oleic, and palmitic acids. Analysis of HPLC-APCI-DAD-MS/MS indicated the presence of lutein, zeaxanthin, and β-carotene, highlighting the nutritional and functional potentials of the extracts. Although no antibacterial activity was observed against *S. aureus* and *E. coli*, the ready-to-use extracts obtained via SFE-NHSolv exhibit significant potential for applications in the food, nutraceutical, and cosmetic industries as antioxidants. The environmental impact assessment demonstrated that SFE-NHSolv is a greener extraction method compared to SOX, reinforcing the sustainable valorization of silk industry byproducts in line with circular economy principles. Overall, this study advances green extraction methods and showcases *B. mori* pupae as a promising sustainable biomass source. Future work should explore lipidomics, extract bioactivity, detailed composition, and protein recovery to support a full biorefinery approach for *B. mori* pupae.

From a future perspective, initial efforts should focus on expanding the chemical characterization of minor lipid constituents, including sterols and tocopherols, as well as investigating the bioavailability, stability, and functional performance of *B. mori* lipids and carotenoids in real systems. Subsequent studies may evaluate process optimization at pilot scale, followed by technoeconomic analyses and life-cycle assessments to assess the feasibility of industrial implementation of SFE-NHSolv. In a more advanced stage, integrating lipid extraction with protein recovery, chitin/chitosan isolation, and other valorization routes could enable a complete biorefinery framework, maximizing both the economic and environmental benefits associated with *B. mori* pupae. Overall, this work lays the foundation for the development of advanced green extraction strategies and highlights the potential of *B. mori* pupae as a sustainable and multifunctional biomass.

## Supplementary Material



## Notes

GC–MS chromatograms of SFE-NHSolv and SOX extracts; fatty acid methyl ester profiles with RT, molecular formula, exact mass, fragment ions (*m*/*z*), and similarity scores; Mann–Whitney U test results for fatty acid composition; HPLC–DAD chromatograms (280 nm) of saponified SOX extracts (B+FD and FD); and antibacterial inhibitory halo images of SOX and SFE-NHSolv extracts (PDF)
