# Supplementary material for: Bombyx mori Pupae as a Novel Ingredient from an Underutilized Sericulture Product: A Dual Approach Based on Sustainable Extractions and Sample Pretreatment Strategies
Source: ACS Sustain Chem Eng. 2026 Jan 9;14(3):1487–501. doi: 10.1021/acssuschemeng.5c10626 (PMC12849045; doi:10.1021/acssuschemeng.5c10626)
Supplement: Supplementary file 1 [file sc5c10626_si_001.pdf]

Supplementary material

Bombyx mori pupae as a novel ingredient from an underutilized sericulture product: A dual-approach based on sustainable extractions and sample pre-treatment strategies

Guilherme Dallarmi Sorita[1,2], Luca Tassoni[3], Alessio Saviane[3], Alejandro Cifuentes[1], Elena Ibáñez[1], Luana Cristina dos Santos[1,*]

[1]Foodomics Laboratory, Institute of Food Science Research (CIAL) (CSIC-UAM), Nicolás Cabrera 9, 28049 Madrid, Spain

[2]Department of Chemical and Food Engineering, Federal University of Santa Catarina, Florianópolis 88040-900, Santa Catarina, Brazil

[3]Council for Agricultural Research and Economics – Research Centre for Agriculture and Environment

*Corresponding author: *luana.dsantos@csic.es*

Summary:

Number of pages: 7

Number of figures: 3

Number of tables: 2

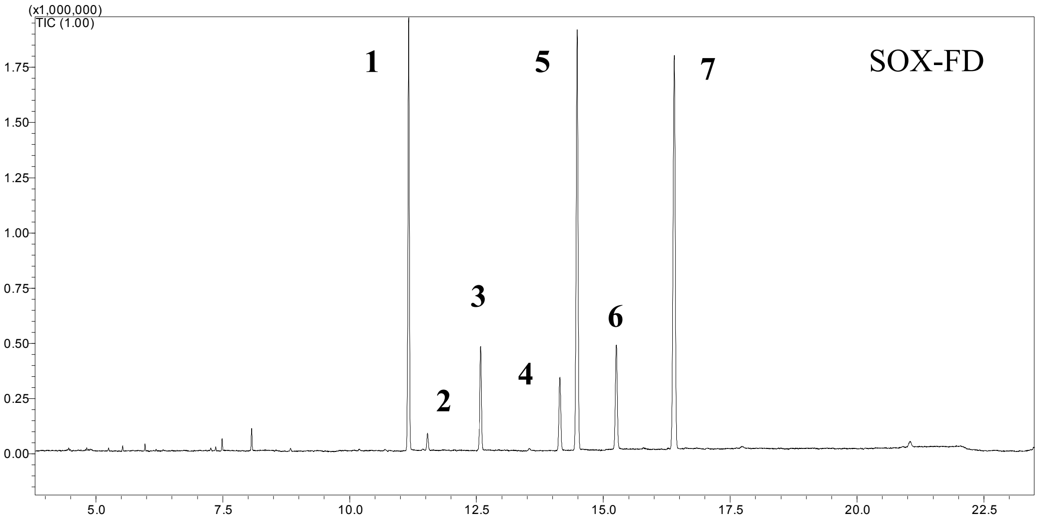
(x1,000,000)
TIC (1.00)
1.75
1.50
1.25
1.00
0.75
0.50
0.25
0.00
5.0
7.5
10.0
12.5
15.0
17.5
20.0
22.5
1
2
3
4
5
6
7
SOX-FD

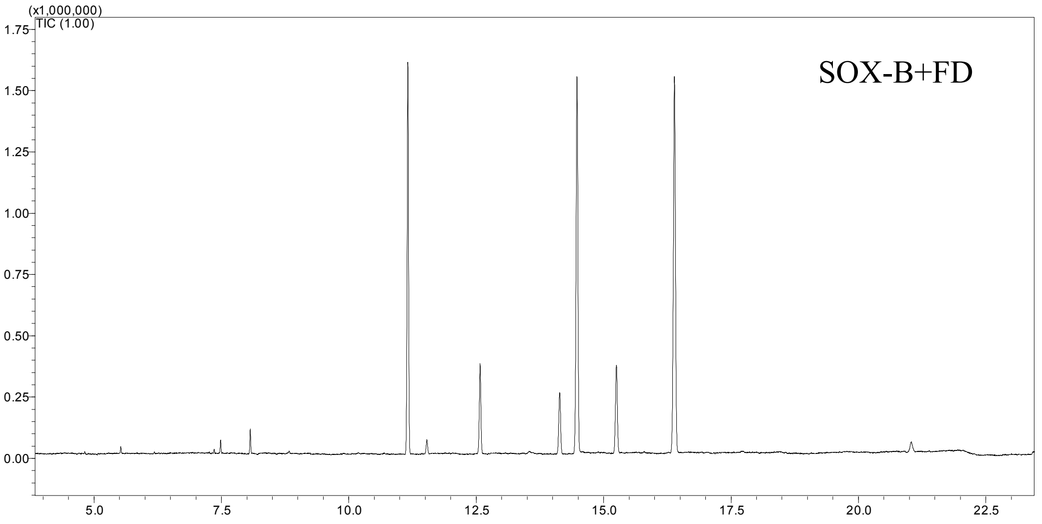
(x1,000,000)
TIC (1.00)
1.75
1.50
1.25
1.00
0.75
0.50
0.25
0.00
5.0
7.5
10.0
12.5
15.0
17.5
20.0
22.5
SOX-B+FD

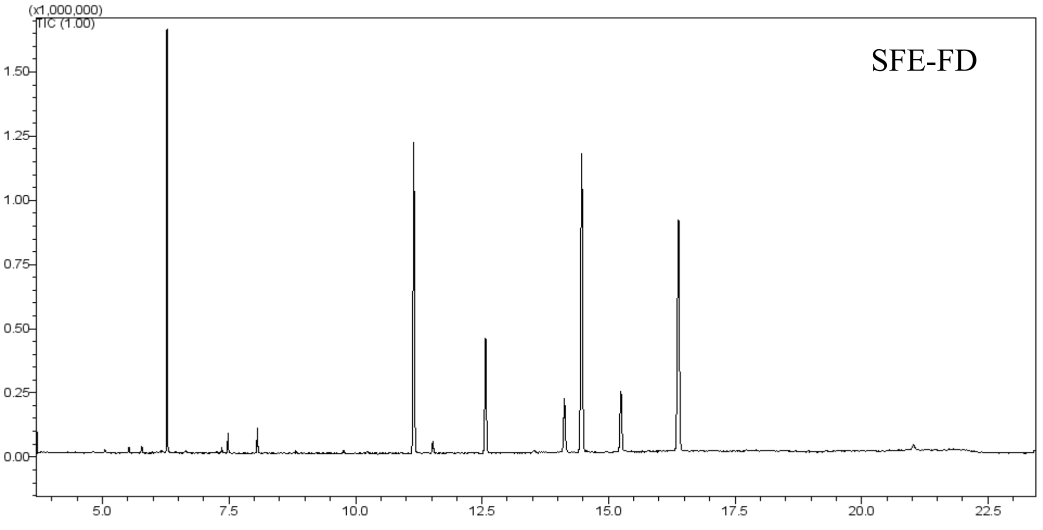
(x1,000,000)
TIC (1.00)
1.50
1.25
1.00
0.75
0.50
0.25
0.00
5.0
7.5
10.0
12.5
15.0
17.5
20.0
22.5
SFE-FD

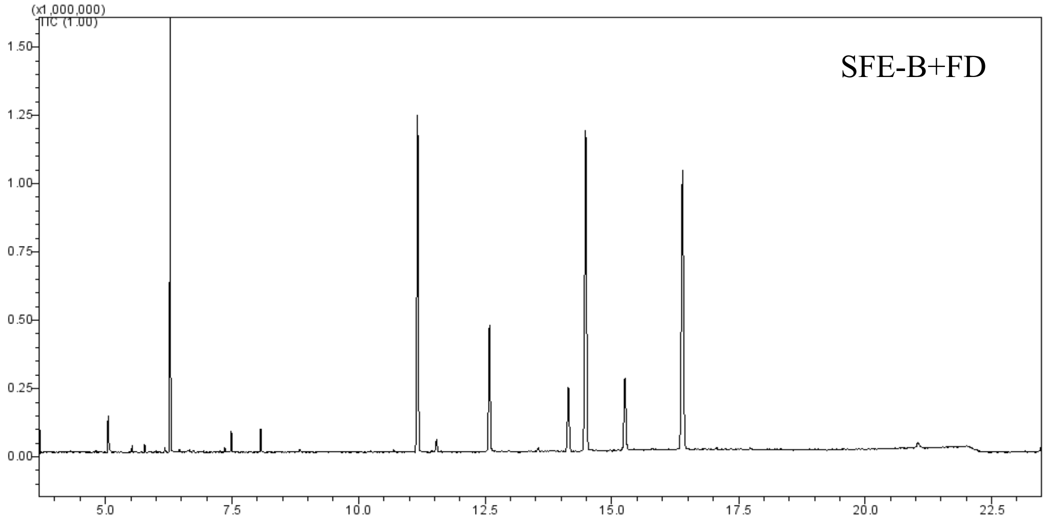

Figure S1. GC-MS chromatograms of SFE-NHSolv and SOX extracts from *Bombyx mori* pupae pretreated by blanching followed by freeze-drying (B+FD) and by freeze-drying only (FD). Peak numbers as in **Table S1**.

Table S1. Fatty acids (as methyl esters) profile of SFE-NHSolv and SOX extracts by GC-MS, detailing the fatty acids (as methyl ester), retention time (RT, min), molecular formula, exact mass, key fragment ions (*m/z*) and similarity (% Sim.) according to updated library databases (NIST, WILEY).

ID	Fatty acids (as methyl esters)	RT (min)	Molecular formula	Exact Molecular Mass	Main fragment ions (*m/z*)	% Sim.
**1**	Palmitic acid	11.16	$C_{16}H_{32}O_2$	256.24	74.05, 87.05, 43.10, 55.05	94
**2**	Palmitoleic acid	11.54	$C_{17}H_{32}O_2$	268.24	55.05, 41.05, 69.10, 43.05	91
**3**	Margaric acid*	12.58	$C_{17}H_{34}O_2$	270.25	74.05, 87.05, 43.05, 55.05	92
**4**	Stearic acid	14.14	$C_{18}H_{36}O_2$	284.27	74.05, 87.05, 43.10, 55.05	92
**5**	Oleic acid	14.48	$C_{18}H_{34}O_2$	282.25	55.05, 69.10, 74.05, 41.10	93
**6**	Linoleic acid	15.26	$C_{18}H_{32}O_2$	280.24	67.05, 81.05, 55.05, 68.10	91
**7**	Linolenic acid	16.40	$C_{18}H_{30}O_2$	278.22	79.05, 67.05, 80.05, 81.05	92

*Internal standard

Table S2. Mean comparison results from Mann Whitney U test for fatty acid profile of *B. mori* pupae extracts (group 1 *vs.* group 2). (NS = groups are not significantly different; * = groups are significantly different at a statistical confidence level of $P \leq 0.1$).

Group 1 vs. Group 2	Palmitic	Palmitoleic	Stearic	Oleic	Linoleic	Linolenic
**SOX-FD vs. SOX-B+FD**	NS (p = 0.2453)	NS (p = 0.8852)	NS (p = 0.3123)	* (p = 0.0265)	NS (p = 0.1939)	* (p = 0.0265)
**SOX-FD vs. SFE-NHSolv-FD**	* (p = 0.0518)	* (p = 0.0952)	* (p = 0.0952)	* (p = 0.0952)	* (p = 0.0952)	* (p = 0.0952)
**SOX- FD vs. SFE-NHSolv-B+FD**	* (p = 0.0304)	* (p = 0.0304)	* (p = 0.0265)	* (p = 0.0265)	* (p = 0.0265)	* (p = 0.0265)
**SOX-B+FD vs. SFE-NHSolv-FD**	* (p = 0.0518)	* (p = 0.0952)	* (p = 0.0952)	* (p = 0.0952)	* (p = 0.0952)	* (p = 0.0952)
**SOX-B +FD vs. SFE-NHSolv-B+FD**	* (p = 0.0304)	* (p = 0.0304)	* (p = 0.0265)	* (p = 0.0304)	* (p = 0.0265)	* (p = 0.0265)
**SFE-NHSolv-FD vs. SFE-NHSolv-B+FD**	NS (p = 0.2159)	* (p = 0.0952)	* (p = 0.0952)	* (p = 0.0952)	* (p = 0.0952)	* (p = 0.0952)

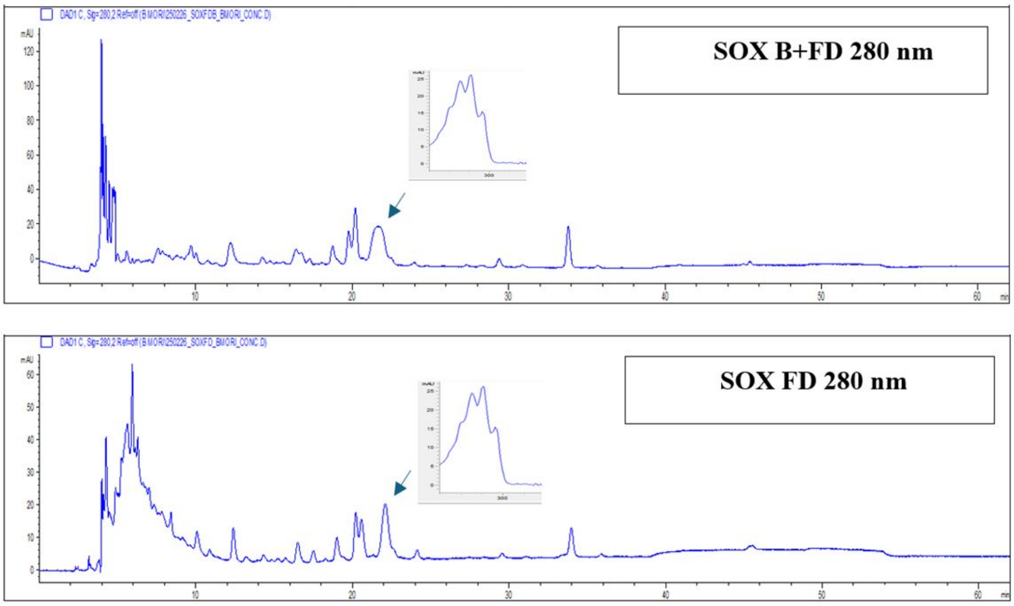

Figure S2. HPLC-DAD chromatograms at 280 nm of saponified SOX extracts from *B. mori* pupae pretreated by blanching followed by freeze-drying (SOX B+FD) and by freeze-drying (SOX FD).

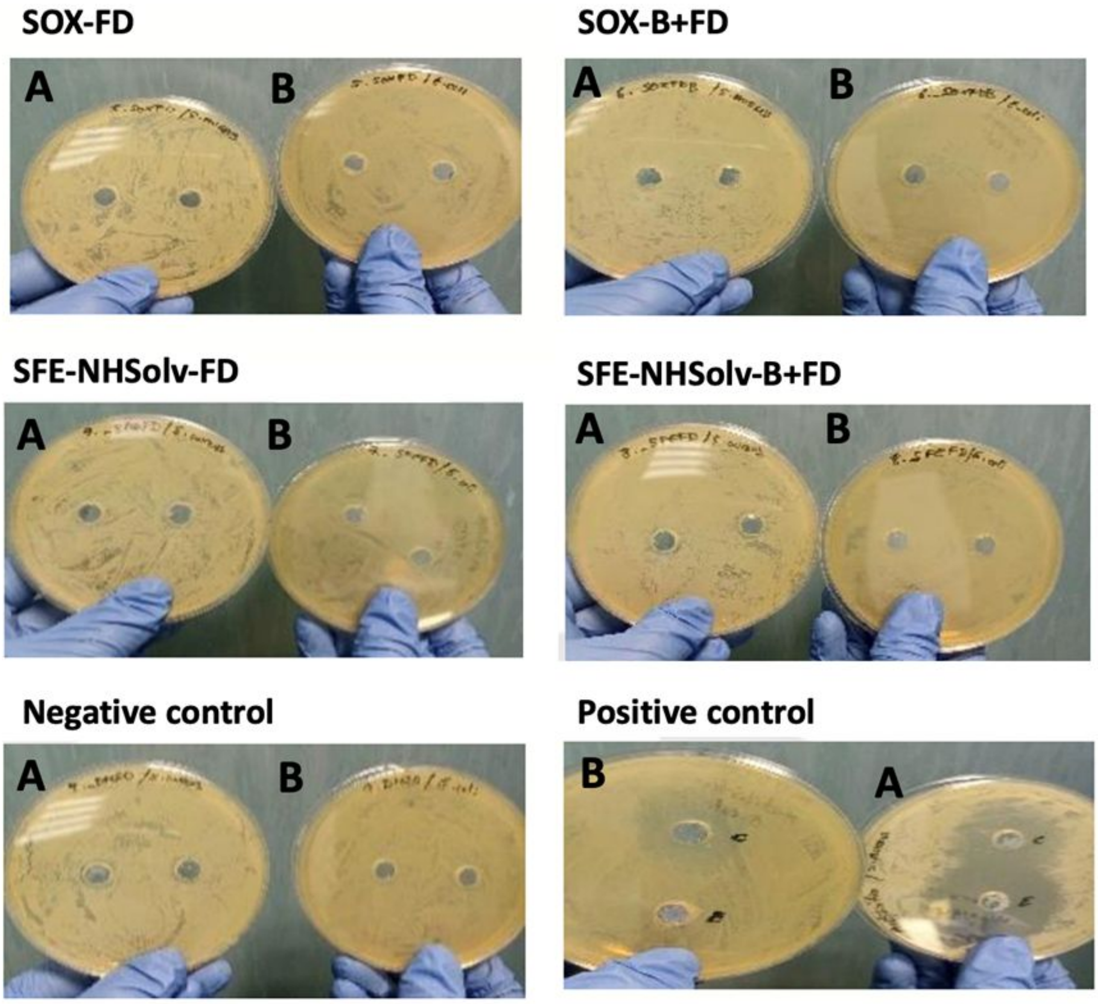

Figure S3. Antibacterial activity of SOX and SFE-NHSolv considering freeze-drying (FD) and blanching+freeze drying (B+FD) as pretreatments for (A) *Staphylococcus aureus* and (B*) Escherichia coli* at 10 mg mL-1. Legend: C: chloramphenicol and E: erythromycin both at 0.1 mg mL-1.
